# Overexpression of DSCR1 prevents proliferation and predicts favorable prognosis in colorectal cancer patients

**DOI:** 10.1186/s12957-021-02212-7

**Published:** 2021-04-07

**Authors:** Wen-Xiang Li, Jia-Jia Zheng, Gang Zhao, Chen-Tao LYU, Wei-Qi Lu

**Affiliations:** 1 Department of General Surgery, Shanghai Public Health Clinical Center, Fudan University, Shanghai, China; 2 Department of General Surgery, Zhongshan Hospital, Fudan University, Shanghai, China

**Keywords:** Colorectal cancer, DSCR1, Immunohistochemistry, Prognosis, Oncomine

## Abstract

**Objectives:**

Down syndrome critical region 1 (DSCR1) is associated with carcinogenesis and tumor growth in several types of malignancy. However, little is known about the role of DSCR1 in CRC progression. The present study aimed to elucidate the clinicopathological significance, prognostic, and function roles of DSCR1 in CRC.

**Methods:**

Firstly, we analyzed DSCR1 expression in 58 paired CRC samples and Oncomine database. Then, we analyzed DSCR1 expression in two independent CRC cohorts (test cohort: *n* = 70; validation cohort: *n* = 58) and tested its overall survival (OS) by Kaplan-Meier survival analyses. Finally, we overexpressed DSCR1 in two CRC cell lines DLD1 and LoVo and analyzed its effect on cell cycle and senescence.

**Results:**

DSCR1 expression was significantly decreased in CRC samples and associated with clinicopathologic features of CRC patients, such as tumor size, lymph node metastasis, and TNM stage. CRC patients with low expression of DSCR1 had shorter overall survival (OS). Kaplan-Meier survival analyses showed that the expression of DSCR1 was significant factor for OS in both cohorts. Multiple Cox regression analysis showed that DSCR1 expression was an independent prognostic marker for OS in test cohort. Overexpression of DSCR1 isoform 4 (DSCR1-4) increased p21, p16, p-NFAT1, and p-NFAT2, while decreased CDK2, CDK4, and Cyclin D1 in CRC cells. In addition, overexpression of DSCR1-4 prevented proliferation and colony formation, and induced senescence in vitro. Moreover, overexpression of DSCR1-4 inhibited tumor growth and tumor angiogenesis in vivo.

**Conclusions:**

Our study found high expression of DSCR1 contributes to favorable prognosis of CRC patients and prevents cell cycle and proliferation of CRC cells, indicating a critical tumor suppressive role in CRC progression.

**Supplementary Information:**

The online version contains supplementary material available at 10.1186/s12957-021-02212-7.

## Background

Colorectal cancer (CRC) is the third most frequently diagnosed cancer and one of the leading death causes of cancer patients worldwide [1]. Although most CRC patients could be fully cured underwent curative resection if diagnosed at early stage, there are about 20–45% of patients with surgery developed recurrence or metastasis [2]. The prognosis of metastatic CRC patients is poor with 1-year survival rate of 36% [3]. With the development of science and technology, the diagnosis rate of early stage CRC cancers has been increasing, leading to decreased mortality of CRC patients. Generalized screening of older population, such as people after 50 years of age, is an effective way to find CRC patients with earlier staged cancers [4]. For persons at risk for young-onset CRC (younger than 50 years), CRC alarm symptoms, family history, and genetic syndromes should be particularly concerned [5]. In addition, control of dietary and lifestyle, such as reducing intake of high-energy snack foods and high-energy drinks, was also reported to associate to the incidence of CRC [6]. However, these approaches are far from improving the damage of this global malignancy. Further underlying mechanisms for the initiation and development of CRC need to be clarified urgently.

Down syndrome critical region gene 1 (DSCR1), also known as regulator of calcineurin 1 (RCAN1), locates on the region 21q22.1-q22.2 and comprises seven exons, four of which (exons 1–4) are alternative first exons [7]. It is reported that DSCR1 interacts with calcineurin A and inhibits calcineurin-dependent signaling pathways, affecting several pathological processes such as Alzheimer disease progression [8], angiogenesis [9, 10], cardiac hypertrophy [11], and myocardial damage [12]. Interestingly, recent several studies showed that DSCR1 played a tumor suppressive role in several types of solid tumors, such thyroid cancer, liver cancer, and lung cancer [13–15]. However, the function of DSCR1 in colon cancer is controversial. It has been reported that syngeneic DSCR1 knockout mice has hyperactivated calcineurin and precocious endothelial apoptosis, which leads to inhibiting formation of an effective tumor vasculature and suppressing tumorigenesis of colon cancer [16]. Another study indicated that DSCR1 may function as a tumor suppressor in early stage transformation of colon cancer by negatively regulating PPARgamma signaling [17]. Therefore, it is important to further investigate the direct role of DSCR1 in colon cancer progression. Here, we investigated the expression of CRC and evaluated its prognostic significance and function in CRC.

## Methods

### Tissue samples and immunohistochemistry (IHC)

This study involved two independent cohorts of patients with CRC: test cohort (70 CRC patients) and validation cohort (58 CRC patients). The test cohort contains 70 patients with primary CRC who underwent surgery at Zhongshan Hospital between 2006 and 2011, and only paraffin-embedded specimens of CRC tissues were obtained from the pathology department. The validation cohort contains 58 patients with primary CRC who underwent surgery at Zhongshan Hospital between 2011 and 2014, and both paraffin-embedded specimens of CRC tissue and para-cancerous tissue were obtained from the pathology department. The study was approved by the Research Ethics Committee of Zhongshan Hospital, Fudan University (Shanghai, China), and written informed consent was obtained from all patients. All specimens were handled and anonymized according to the ethical standards of Fudan University. All patients did not receive neoadjuvant therapy prior to surgery. Patients without follow-up information were not included in our study. TNM stage of CRC patients was defined according to the 6th edition of the TNM staging system of the American Joint Committee on Cancer (AJCC)/International Union Against Cancer (UICC). Tumor tissues were fixed with 10% formalin and embedded in paraffin before prepared for tissue microarray. Briefly, 1-mm cores were taken from intratumoral tissue and serial sections (4-μm thick) were placed on slides coated with 3-aminopropyltriethoxysilane. The IHC analysis was also conducted as described previously [18]. Immunostaining scores were independently evaluated by two pathologists who were blinded to the clinical outcome. The sections were incubated with primary goat anti-human DSCR1 antibody (D6694, 1:50, Sigma) at 4 °C overnight, and then secondary antibody within 30 min. The average sum of integrated optical density (IOD) of each sample was calculated using ImageJ software.

### Oncomine database analysis

Gene expression changes were analyzed in the TCGA microarray dataset of the Oncomine website with colorectal tumor and normal colorectal tissues (www.oncomine.org, Compendia biosciences, Ann Arbor, MI, USA). The threshold search criteria used in the study were a *p*-value of < 0.01, a fold change of > 2.

### Cell culture

The human CRC cell lines DLD1 and LoVo were purchased from the American Type Culture Collection. Both cell lines were cultured in RPMI-1640 medium (Gibco; Thermo Fisher Scientific, Inc.), supplemented with 10% FBS (Invitrogen; Thermo Fisher Scientific, Inc.), 100 U/ml penicillin and 100 g/ml streptomycin, and maintained at 37 °C in a 5% CO_2_ humidified atmosphere.

### Construction of DSCR1-1 and DSCR1-4 overexpressing CRC cell lines

The DSCR1-1 open reading frame sequence (NM_004414.7) and DSCR1-4 open reading frame sequence (NM_203418.2) were constructed and cloned into a lentiviral expression vector pWPXL (Addgene), respectively. The recombinant vector was co-transfected into 293T cells alongside packaging plasmid psPAX2 (Addgene) and envelope plasmid pMD2.G (Addgene) using Lipofectamine® 2000 reagent (Invitrogen; Thermo Fisher Scientific, Inc.). The empty pWPXL vector was used as a control for the infection. After virus collection, DLD1 and LoVo cells were subsequently infected with the lentivirus in the presence of 2 μg/ml polybrene (Sigma-Aldrich; Merck KGaA).

### Western blotting

Total proteins of control cells and DSCR1 overexpression cells were extracted using protein extraction reagent (RIPA; Thermo Fisher Scientific, Inc.). Equal amounts of total proteins were separated by using 10% SDS-PAGE. The separated proteins were subsequently transferred onto PVDF membranes (Millipore) and blocked with PBST (pH 7.4) containing 5% non-fat milk and 0.1% Tween-20. The membranes were then incubated with primary antibodies (anti-HSP90, #4874, CST, 1:1000; anti-p21, ab109199, Abcam, 1:1000; anti-p16, #80772, CST, 1:1000; anti-pNFAT1, Catalog # 44-944G, Thermo Fisher, 1:1000; anti-pNFAT2, Catalog # 679340, NOVUS, 1:1000; anti-CDK2, #18048, CST, 1:1000; anti-CDK4, #12790, CST, 1:1000; anti-cyclin D1, #55506, CST, 1:1000; anti-DSCR1, Sigma-Aldrich WH0001827M3, 1:1000) overnight at 4 °C. Then, the membranes were further incubated with HRP-conjugated secondary antibodies at room temperature. The bands were obtained by using an ECL reagent (Pierce; Thermo Fisher Scientific, Inc.) and a ChemiDoc MP Imaging System (Bio-Rad Laboratories, Inc.).

### CCK-8 assays

Cell viability of DLD1 and LoVo was determined by CCK-8 assays. Indicated cells (20 μl; 3000/well) were seeded in a 96-well plate, and then 20 μl of CCK-8 was added into each well 1, 2, 3, and 4 days later. OD450 was measured using an enzyme microplate reader.

### Colony formation assay

For the plate colony formation assay, DLD1 and LoVo cells were seeded into six-well plates at 37 °C with 5% CO_2_. After 10–14 days culture, the plates were washed with PBS, fixed with 4% paraformaldehyde at room temperature and stained with 1.5% crystals violet (Sigma-Aldrich; Merck KGaA).

### β-galactosidase (SA-β-Gal) staining

The senescence-related SA-β-Gal staining was performed by using a β-Gal Staining Kit (Thermo Fisher Scientific; catalog number: K146501). Briefly, indicated cells were seeded in six-well plates, and the plates were washed with PBS for 2 times before adding β-galactosidase fixed solution. The cells were fixed with 1 mL β-galactosidase fixed solution at room temperature for 15 min. Then, the plates were washed for 3 times with PBS and added with 1 mL dyeing liquid (10 μL β-galactosidase staining fluid A, 10 μL β-galactosidase staining fluid B, 930 μL β-galactosidase staining fluid C, and 50 μL X-Gal solution) at 37 °C overnight. Finally, the plates were observed under the inverted microscope. The calculation method for positive rate of SA-β-Gal staining was as follows: 5 views were randomly selected from each well under × 200 magnification, and the percentage of positive cells was calculated as percentage of total cell number.

### Xenograft model

Male BALB/c-nude mice (4 weeks of age, 5 mice per group) were subcutaneously injected with 2 × 10^6^ control LoVo cells and DSCR1-4 overexpression LoVo cells into the right flanks, respectively. Tumors were measured with calipers and calculated with the formula: Volume (mm^3^) = [width^2^ (mm^2^) × length (mm)]/2. Finally, tumors were dissected, pictured, and weighed.

### Statistical analysis

Differences among variables were assessed by *χ*^2^ analysis or two-tailed Student *t* test. Kaplan-Meier analysis was used to assess survival. Log-rank tests were used to compare survival of patients between subgroups. Multivariate analyses were performed by multivariate Cox proportional hazard regression model. Data were presented as mean ± SEM. Differences were considered to be statistically significant for *p* < 0.05.

## Results

### DSCR1 was downregulated in CRC tissues

**Fig. 1:**
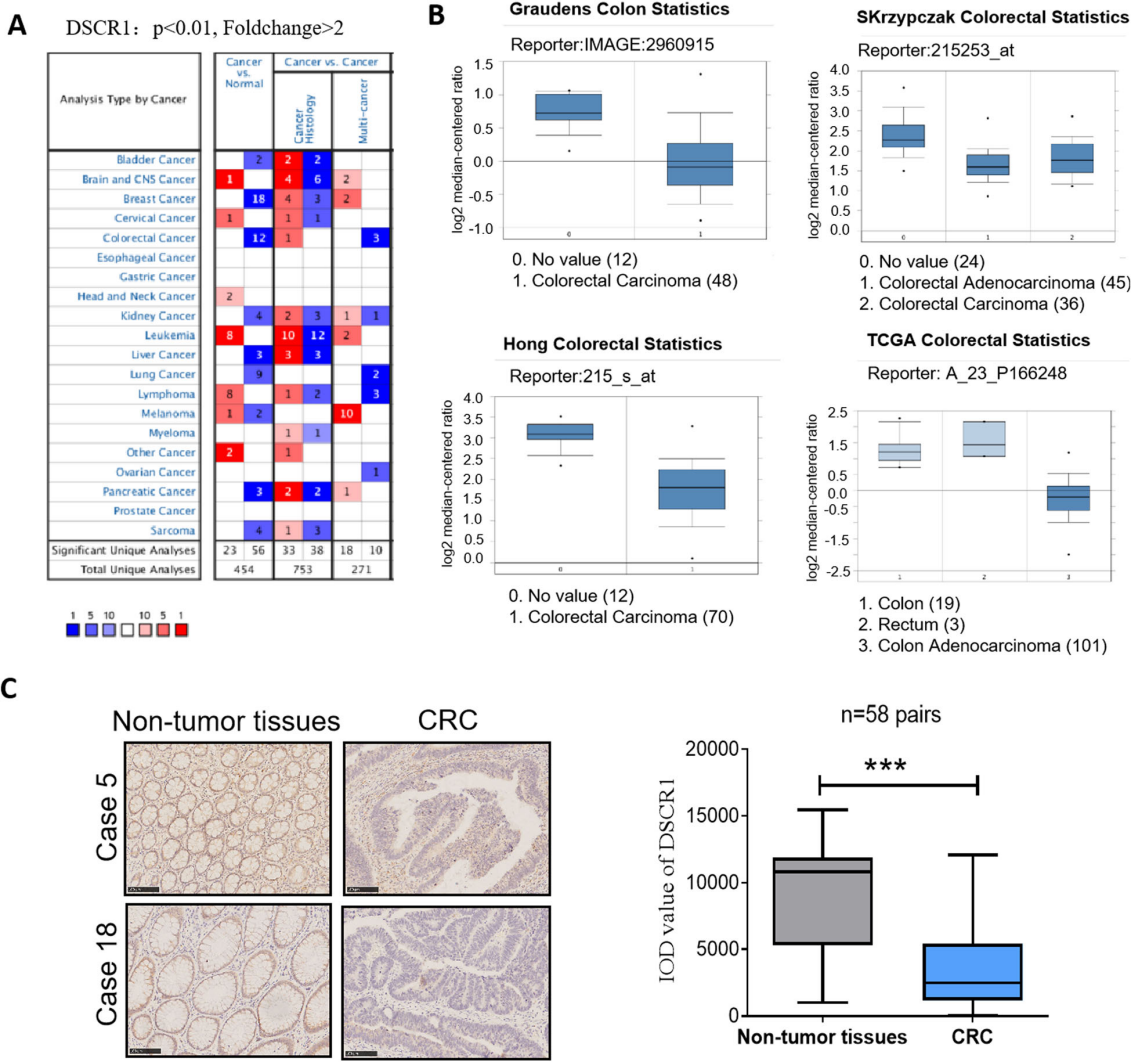
DSCR1 is downregulated in CRC tissues. **a** Summary of GRIK1 expression in Oncomine datasets. **b** Four representing Oncomine datasets. **c** IHC staining of DSCR1 in 58 pairs of CRC tissues (CRC) and normal tissues. Two-tailed Student *t* test, ***p < 0.001; scale bar = 100 μm

To investigate the role of DSCR1 in CRC progression, we firstly analyzed expression of DSCR1 in Oncomine database. The results showed that DSCR1 level was significantly decreased in CRC tissues compared to normal colon tissues in Oncomine dataset (Fig. 1a, b). We further validated the expression level of DSCR1 in 58 pairs of CRC specimens (tumor and corresponding non-tumor tissues) by IHC analyses. The results showed that the protein level of DSCR1 was significantly downregulated in CRC tissues (Fig. 1c).

### Low expression of DSCR1 significantly correlated with clinicopathological significance of CRC patients

Then, we performed IHC analysis for DSCR1 using a tissue microarray (TMA) as a test cohort, which contained 70 CRC tissue samples. Expression of DSCR1 was observed in 31 patients with CRC (44.3%). The correlations between DSCR1 expression and the clinicopathological factors of the patients are presented in Table 1. As the data showed, the expression level of DSCR1 was significantly correlated with the tumor size (*p* = 0.018), lymph node metastasis (*p* = 0.013), histological grade (*p* = 0.008), node stage (*p* = 0.016), and TNM stage (*p* = 0.037) of CRC patients (Table 1). We next performed IHC analysis for DSCR1 using another TMA as a validation cohort, which contained 58 CRC tissue samples. Expression of DSCR1 was observed in 23 patients with CRC (39.7%) (Table 1). The expression level of DSCR1 was also significantly correlated with the tumor size (*p* = 0.046), lymph node metastasis (*p* = 0.016), and TNM stage (*p* = 0.001) of CRC patients (Table 1).

**Table 1:** Relationship between DSCR1 expression and clinicopathologic features of CRC patients

Characteristics	Case number	Test cohort			Case number	Validation cohort		
		DSCR1 negative	DSCR1 positive	*p* value		DSCR1 negative	DSCR1 positive	*p* value
**Age**				0.141				0.271
≤ 60	28	19	9		20	10	10	
>60	42	20	22		38	25	13	
**Sex**				0.808				0.417
male	41	22	19		33	18	15	
female	29	17	12		25	17	8	
**Location**				1.000				0.592
colon	38	21	17		32	18	14	
rectum	32	18	14		26	17	9	
**Tumor side**				0.9003				0.524
Left	32	13	19		25	12	13	
Right	38	16	22		33	19	14	
**Tumor size**				***0.018***				***0.046***
≤ 6 cm	36	15	21		31	15	16	
>6 cm	34	24	10		27	20	7	
**Tumor differentiation**				0.091				0.059
well/moderate	32	14	18		28	13	15	
poor/undifferentiated	38	25	13		30	22	8	
**Lymph node metastasis**				***0.013***				***0.016***
no	53	25	28		34	16	18	
yes	17	14	3		24	19	5	
**Histological grade**				***0.008***				0.593
I/II	20	6	14		27	15	12	
III	50	33	17		31	20	11	
**Node stage**				***0.016***				0.111
N0	41	17	24		27	13	14	
N1	23	17	6		19	12	7	
N2	6	5	1		12	10	2	
**TNM stage**				***0.037***				***0.001***
I	5	2	3		15	1	14	
II	35	15	20		18	11	7	
III	30	22	8		19	17	2	

### DSCR1 expression exhibited prognostic value for CRC patients

**Fig. 2:**
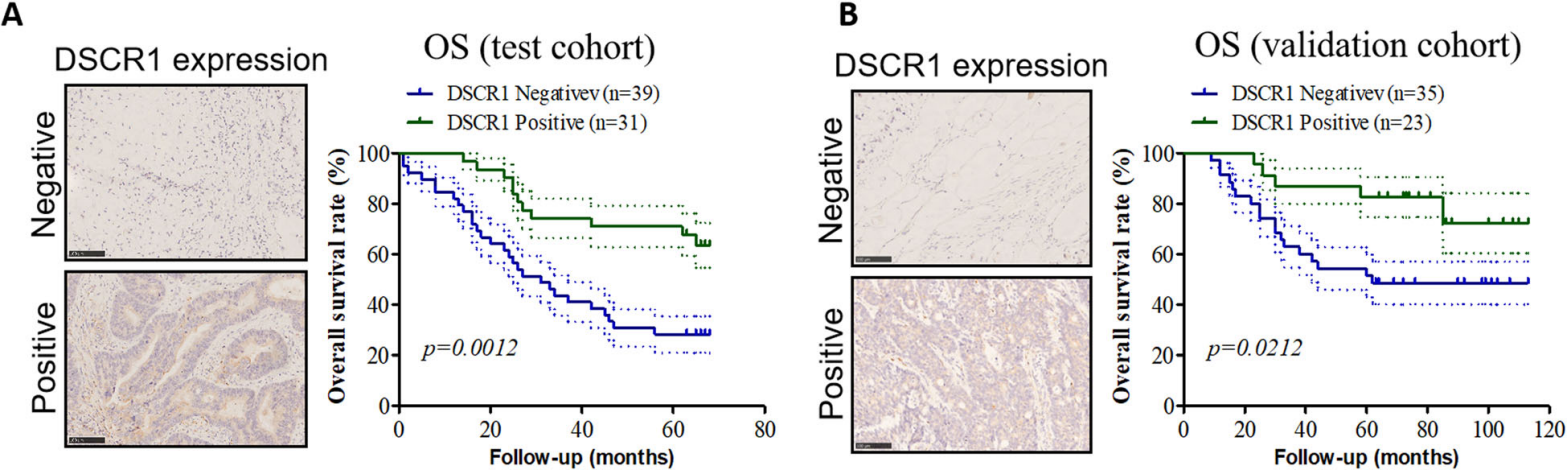
DSCR1 expression exhibited prognostic value for CRC patients. **a** Kaplan-Meier analysis of the correlation between DSCR1 expression and the overall survival of CRC patients in the test cohort (*n* = 70). Log-rank tests were used to determine statistical significance. Scale bar = 100 μm. **b** Kaplan-Meier analysis of the correlation between DSCR1 expression and the overall survival of CRC patients in the validation cohort (*n* = 58). Log-rank tests were used to determine statistical significance. Scale bar = 100 μm

We further analyzed the prognostic value of DSCR1 expression for CRC patients in test and validation cohort, respectively. The follow-up period for patients in test cohort ranged from 1 to 68 months. During the follow-up period in test cohort (70 patients), 39 patients died. Using the Kaplan-Meier method, our results showed that negative expression of DSCR1 was significantly associated with poor OS rates in the test cohort (*p* = 0.0012; Fig. 2a). For the validation cohort, the follow-up period for patients in test cohort ranged from 9 to 113 months. During the follow-up period in test cohort (58 patients), 23 patients died. Similarly, negative expression of DSCR1 was also significantly associated with poor OS rates in the validation cohort (*p* = 0.0212; Fig. 2b).

### DSCR1 expression was an independent prognostic value for CRC patients

To evaluate whether the expression levels of DSCR1 was independent prognostic value for CRC patients in our cohorts, univariate and multivariate analyses using a Cox regression model were applied. As shown in Table 2, tumor size, lymph node metastasis, histological grade, node stage, TNM stage, and DSCR1 expression was responsible for the OS of CRC patients in the test cohort. Multiple Cox regression analysis showed that only tumor size, lymph node metastasis, histological grade, node stage, and DSCR1 expression were independent factors for CRC patients in this cohort. In our validation cohort, as shown in Table 2, tumor size, tumor differentiation, lymph node metastasis, node stage, TNM stage, and DSCR1 expression was responsible for the OS of CRC patients. However, multiple Cox regression analysis showed that only tumor size, tumor differentiation, and lymph node metastasis were independent factors for CRC patients in this cohort. Collecting, the results indicated that the DSCR1 expression appeared to be an independent prognostic factor for CRC patients.

**Table 2:** Univariate and multivariate analysis of variant parameters in CRC patients by Cox regression analysis

Variables	Test cohort			Validation cohort		
	Multivariate			Multivariate		
	Univariate *p value*	HR (95%CI)	*p value*	Univariate *p value*	HR (95%CI)	*p value*
Age: ≤60 vs >60	0.793	NA	NA	0.095	NA	NA
Sex: male vs female	0.342	NA	NA	0.573	NA	NA
Location: colon vs rectum	0.818	NA	NA	0.528	NA	NA
Tumor size: ≤ 6 cm vs >6 cm	***0.000***	2.336 (1.114-4.897)	***0.025***	***0.000***	3.790 (1.321-10.874)	**0.013**
Tumor differentiation: well/moderate vs poor/undifferentiated	0.589	NA	NA	***0.000***	6.426 (2.008-20.570)	**0.002**
Lymph node metastasis: yes vs no	***0.000***	2.256 (1.052-4.837)	***0.037***	***0.000***	10.282 (3.343-31.620)	**0.000**
Histological grade: I / II vs III	***0.001***	3.743 (1.285-10.902)	***0.016***	0.062	NA	NA
Node stage: N0 vs N1 vs N2	***0.000***	1.734 (1.087-2.765)	***0.021***	***0.000***	NA	NA
TNM: I vs II vs III-IV	***0.018***	NA	NA	***0.016***	NA	NA
DSCR1: negative vs positive	***0.001***	0.355 (0.171-0.738)	***0.047***	***0.021***	NA	NA

### Overexpression of DSCR1-4 showed tumor suppressive function in CRC cells in vitro

**Fig. 3:**
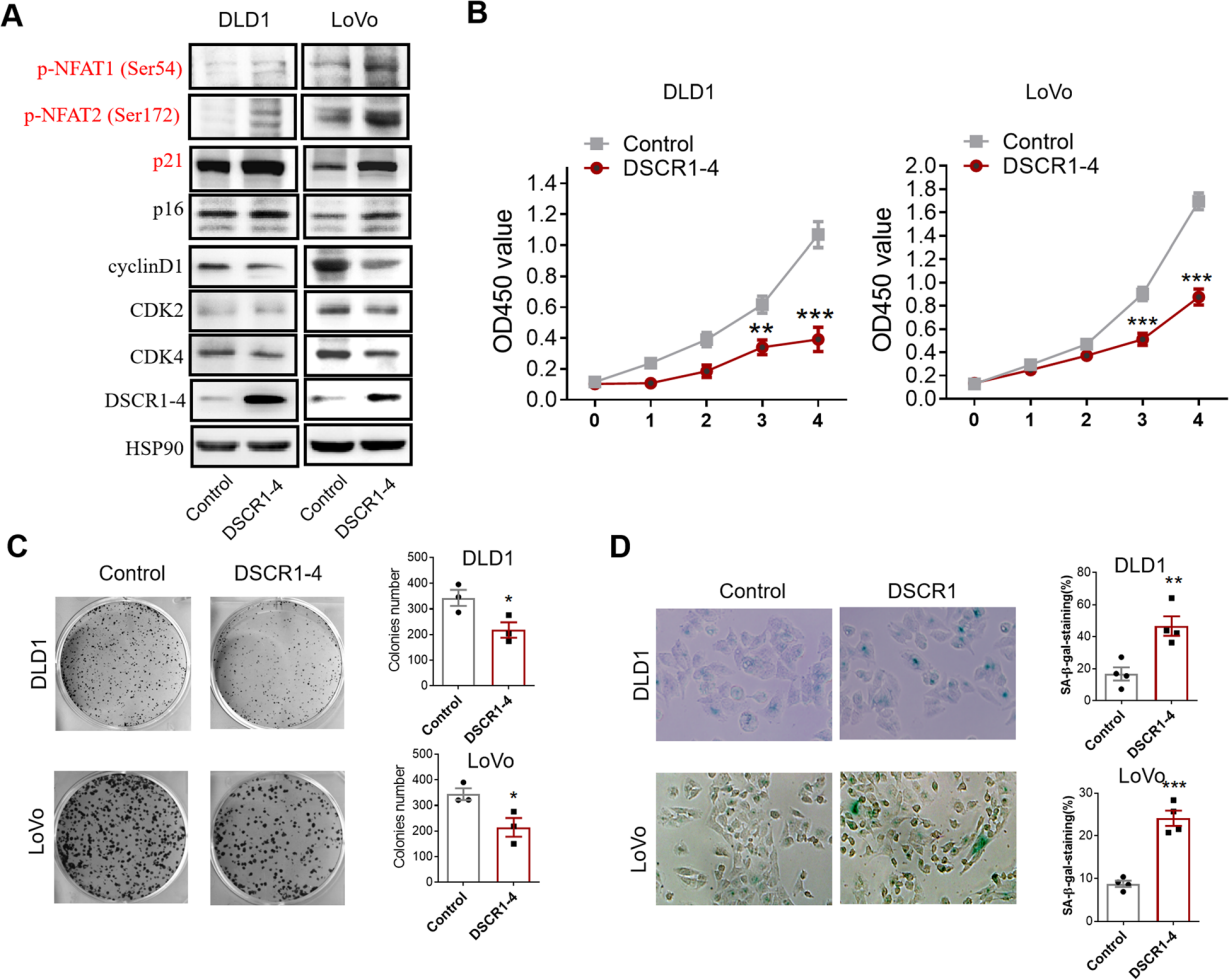
Overexpression of DSCR1-4 showed tumor suppressive function in CRC cells in vitro. **a** Western blot showed that overexpression of DSCR1-4 increased p21, p16, and hyperphosphorylation of NFAT1 and NFAT2, while decreased cyclin D1, CDK2, and CDK4. **b** CCK8 assays showed that overexpression of DSCR1-4 inhibited cell proliferation of CRC cells. Data are mean ± SEM of 5 replicates. **c** Overexpression of DSCR1-4 inhibited cell colony formation of CRC cells. Data are mean ± SEM of 3 replicates. **d** Overexpression of DSCR1-4 induced senescence of CRC cells by SA-β-gal staining. Two-tailed Student *t* test, *p < 0.05, **p < 0.01, ***p < 0.001

DSCR1 has two main transcripts or isoforms DSCR1-1 and DSCR1-4 [19]. To test the functions of these two DSCR1 isoforms in CRC, we overexpressed DSCR1-1 and DSCR1-4 in CRC cells, respectively. We firstly overexpressed DSCR1-1 in LoVo cancer cell line (sFig. 1A). The results showed that overexpression of DSCR1-1 had no significant effect on proliferation and colony formation capability of LoVo cells in vitro (sFig. 1B-C). Then, we further evaluated the effects of DSCR1-4 on CRC cells. After overexpression of DSCR1-4, we firstly determined the expression level of cell cycle-related proteins in DLD1 and LoVo cancer cell lines. Western blot showed that overexpression of DSCR1 upregulated protein level of p21 (p21Waf1/Cip1) and p16 (p16INK4A), while decreased protein level of cyclin D1, CDK2, and CDK4 (Fig. 3a). In addition, overexpression of DSCR1-4 resulted in hyperphosphorylation of NFAT1 and NFAT2 (Fig. 3a), indicating the negative regulation of calcineurin-NAFT signaling in CRC. CCK8 assays and colony formation assays showed that overexpression of DSCR1 inhibited cell proliferation in both DLD1 and LoVo cells (Fig. 3b, c). Moreover, we found that overexpression of DSCR1-4 could induce senescence, which was reflected by SA-β-Gal staining (Fig. 3d). Collectively, these results revealed that DSCR1-4 functioned as a tumor suppressor in CRCR.

### Overexpression of DSCR1-4 inhibited tumor growth and tumor angiogenesis in vivo

**Fig. 4:**
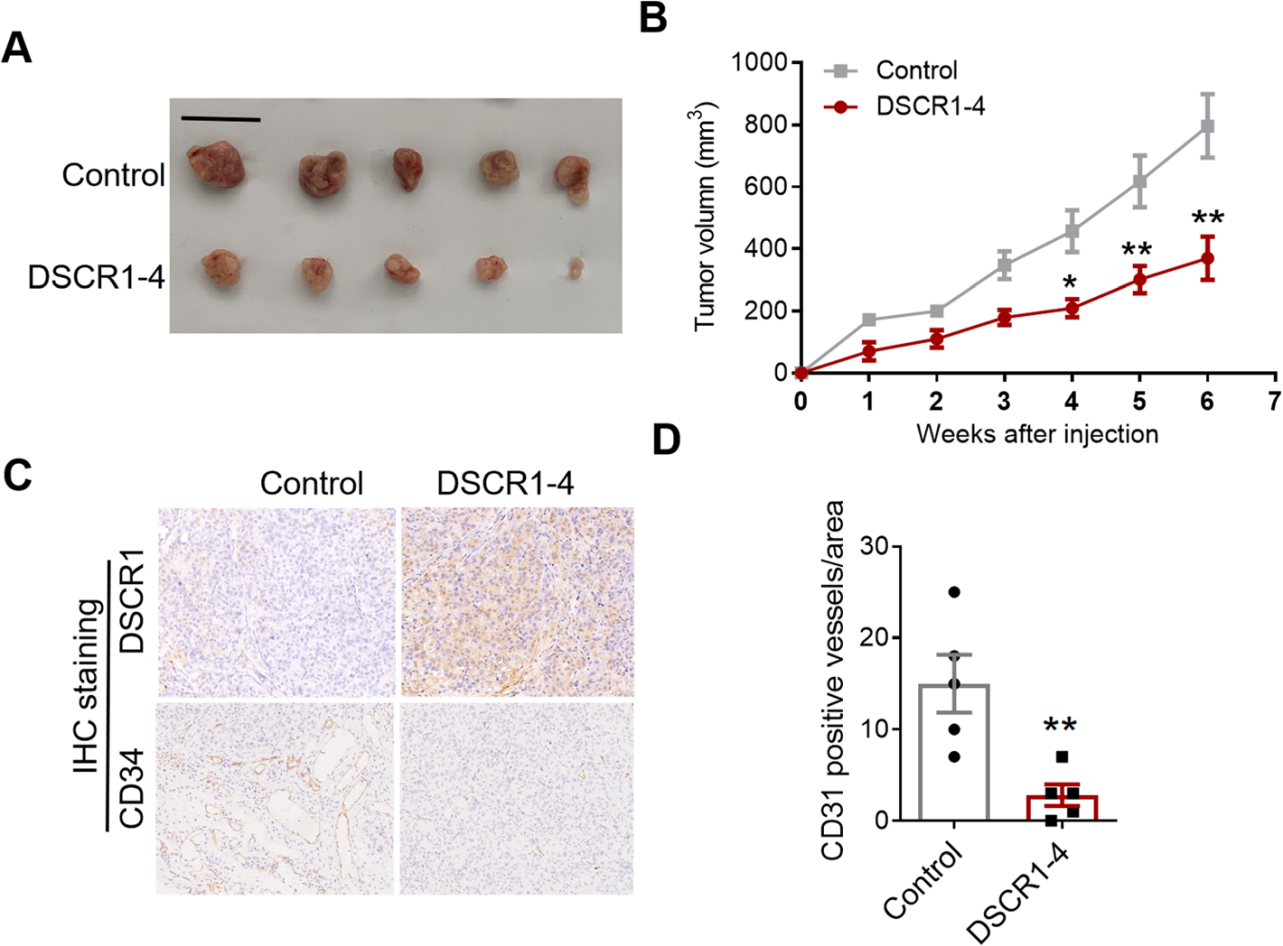
The tumor suppressive effects of DSCR1-4 in CRC in vivo. **a** Images of tumors from nude mice. Scale bars = 1 cm. **b** Tumor growth curves of control and DSCR1-4 overexpression tumors in nude mice. **c** Immunohistochemical staining of DSCR1 and CD31 was performed. **d** Quantification of CD31 positive vessels in control and DSCR1-4 overexpression tumors, respectively. Data are mean ± SEM of 5 replicates. Two-tailed Student *t* test, *p < 0.05, **p < 0.01

To investigate the functional roles of DSCR1-4 in CRC progression in vivo, we injected the control LoVo cells and DSCR1-4 overexpression LoVo cells into nude mice and established a xenograft model. The results showed that overexpression of DSCR1-4 significantly inhibited tumor growth in vivo (Fig. 4a, b). Because DSCR1-4 has been reported to be associated with tumor angiogenesis, we also determined the microvascular density (MVD) by IHC staining of CD31 in tumor tissues from the above xenograft model. The results showed that overexpression of DSCR1-4 reduced the MVD in tumor tissues when compared to that of control tumors (Fig. 4c, d). Taken together, these data supported that DSCR1-4 functioned as tumor suppressor in CRC.

## Discussion

The prognosis of patients with advanced CRC who receive conventional treatment strategies remains poor. The staging system most widely used for colorectal cancer is the American Joint Committee on Cancer (AJCC) TNM system. Although the traditional TNM staging system can indicate prognostication and prediction for adjuvant therapy to some extent, it is still not good enough to individually define a patient’s outcome [20]. For example, TNM staging system cannot distinguish the malignant cancer from the same stage II patients, which causes more than 20% of patients die of recurrent disease [21]. In our both test and validation cohort, even though TNM stage closely associated to prognosis of CRC patients, it cannot be used as an independent prognostic marker for CRC patients. In addition, the stromal factors and immune response are not considered in the current TNM staging system, which are closely related to the outcome of CRC patients [22, 23]. Importantly, several key molecular markers of prognosis have been identified in CRC, such as microRNAs [24], SNORA42 [25], and Aldolase A [26]. However, the association between DSCR1 and clinical significance of CRC is still largely unknown. A study reported that single nucleotide polymorphisms (SNPs) of obesity-related genes including DSCR1 was related to tumor recurrence in stage II/III colon cancer [27]. In this study, we firstly identified DSCR1 as a novel independent prognostic biomarker for CRC patients. We used two independent cohorts of CRC samples to analyze the association between CRC expression and outcomes of CRC patients. In both cohorts, we consistently found that negative expression of DSCR1 was also significantly associated with poor OS rates. Cox regression analysis showed that DSCR1 expression was an independent prognostic marker for CRC patients in our test cohort. However, DSCR1 expression could not serve as an independent prognostic marker for CRC patients in our validation cohort. It may because the small CRC samples enrolled in the validation cohort (only 58 samples). As our results showed in Fig. 2b, DSCR1 expression could predict prognostic value for CRC patients.

It was well studied that DSCR1 could mediate VEGF signaling in vascular endothelial cells, finally participating in angiogenesis and tumor growth [9, 10, 28]. Both in vitro and in vivo studies revealed that VEGF greatly induced DSCR1 in endothelial cells, which negatively regulated the calcineurin-NFAT signaling pathway and acted as an endogenous feedback inhibitor for angiogenesis [9, 10]. Recently, more and more research has focused on the tumor suppressive function of DSCR1 in tumor cells per se. One study reported that isoform 4 of DSCR1 was potently decreased in hepatocellular carcinoma (HCC) samples, which lead to activate of calcineurin/NFAT1 signaling and promote angiogenesis and metastasis of HCC [13]. Another study reported that DSCR1 functioned as a growth and metastasis suppressor of thyroid cancer in part through NFE2L3 [14]. Similarly, DSCR1 was reported to be involved in the development of small cell lung cancer and functioned as a cancer-inhibiting gene for the formation of bone metastases in small cell lung cancer [15]. Our study also showed that DSCR1 expression was significantly decreased in CRC when compared to adjacent non-tumor tissues. Moreover, CRC patients with negative expression of DSCR1 had bigger tumor size, positive lymph node metastasis, and later TNM stage comparing to CRC patients with positive expression of DSCR1. Our in vitro data further confirmed that one major isoform of DSCR1, DSCR1-4, negatively regulated calcineurin-NAFT signaling and inhibited proliferation of CRC cells. All these results indicated the potential tumor suppressive function of DSCR1 in CRC. Calcineurin inhibitors, such as tacrolimus and CsA, can also inhibit calcineurin-NAFT signaling and achieve similar functions of DSCR1. Our study revealed that calcineurin inhibitors may have antitumor activity for patients with CRC who had low RCAN1.4 expression or hyperactivation of calcineurinNFAT1 signaling in tumors. However, further experiments need to be performed to confirm this hypothesis in the future.

## Conclusions

There is still an urgent need to find new tumor suppressor molecules of CRC. Our study, for the first time, found high expression of DSCR1 contributes to favorable prognosis of CRC patients and prevents cell cycle and proliferation of CRC cells, indicating a critical tumor suppressive role in CRC progression.

## Supplementary Information

**Additional file 1: Fig 1.** Overexpression of DSCR1-1 showed no effect on proliferation and colony formation in CRC cells. (**A**). Western blot showed overexpression of DSCR1-1 in LoVo cells. (**B**). CCK8 assays showed that overexpression of DSCR1-1 had no effect on cell proliferation of CRC cells. Data are mean ±SEM of 5 replicates. (**C**). Overexpression of DSCR1-1 had no effect on cell colony formation of CRC cells. Data are mean ±SEM of 4 replicates.

## Data Availability

The data used and/or analyzed during the current study are available from the corresponding author.
