# Supplementary material for: Overexpression of DSCR1 prevents proliferation and predicts favorable prognosis in colorectal cancer patients
Source: World J Surg Oncol. 2021 Apr 7;19:100. doi: 10.1186/s12957-021-02212-7 (PMC8028816; doi:10.1186/s12957-021-02212-7)
Supplement: Supplementary file 1 — Additional file 1: Fig 1. Overexpression of DSCR1-1 showed no effect on proliferation and colony formation in CRC cells. (A). Western blot showed overexpression of DSCR1-1 in LoVo cells. (B). CCK8 assays showed that overexpression of DSCR1-1 had no effect on cell proliferation of CRC cells. Data are mean ±SEM of 5 replicates. (C). Overexpression of DSCR1-1 had no effect on cell colony formation of CRC cells. Data are mean ±SEM of 4 replicates. [file 12957_2021_2212_MOESM1_ESM.zip › Additional file 1.docx]

**sFigure1**


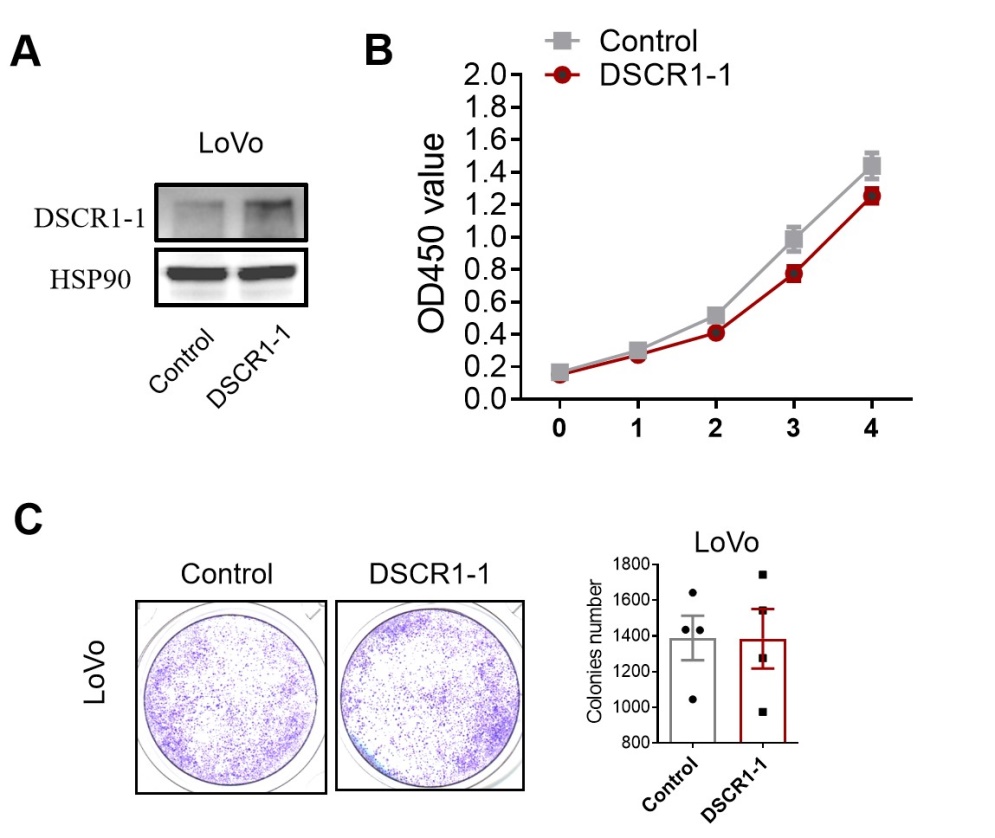


**sFig 1.** Overexpression of DSCR1-1 showed no effect on proliferation and colony formation in CRC cells. (**A**). Western blot showed overexpression of DSCR1-1 in LoVo cells. (**B**). CCK8 assays showed that overexpression of DSCR1-1 had no effect on cell proliferation of CRC cells. Data are mean ±SEM of 5 replicates. (**C**). Overexpression of DSCR1-1 had no effect on cell colony formation of CRC cells. Data are mean ±SEM of 4 replicates.
